# Inflammation-Induced Acute Phase Response in Skeletal Muscle and Critical Illness Myopathy

**DOI:** 10.1371/journal.pone.0092048

**Published:** 2014-03-20

**Authors:** Claudia Langhans, Steffen Weber-Carstens, Franziska Schmidt, Jida Hamati, Melanie Kny, Xiaoxi Zhu, Tobias Wollersheim, Susanne Koch, Martin Krebs, Herbert Schulz, Doerte Lodka, Kathrin Saar, Siegfried Labeit, Claudia Spies, Norbert Hubner, Joachim Spranger, Simone Spuler, Michael Boschmann, Gunnar Dittmar, Gillian Butler-Browne, Vincent Mouly, Jens Fielitz

**Affiliations:** 1 Experimental and Clinical Research Center (ECRC), a Cooperation between Max Delbrück Center and Charité Universitätsmedizin Berlin, Campus Buch, Berlin, Germany; 2 Charité Universitätsmedizin Berlin, Campus Virchow and Campus Mitte, Anesthesiology and Operative Intensive Care Medicine, Berlin, Germany; 3 Max Delbrück Center for Molecular Medicine, Berlin, Germany; 4 Universitätsmedizin Mannheim, Mannheim, Germany; 5 Charité Universitätsmedizin Berlin, NeuroCure Clinical Research Center, Berlin, Germany; 6 Charité Universitätsmedizin Berlin, Department of Endocrinology, Diabetes and Nutrition, Berlin, Germany; 7 Institut de Myologie, Institut national de la santé et de la recherche médicale, and L’Université Pierre et Marie Curie Paris, Paris, France; 8 Charité Universitätsmedizin Berlin, Campus Virchow, Cardiology, Berlin, Germany; D’or Institute of Research and Education, Brazil

## Abstract

**Objectives:**

Systemic inflammation is a major risk factor for critical-illness myopathy (CIM) but its pathogenic role in muscle is uncertain. We observed that interleukin 6 (*IL-6*) and serum amyloid A1 (*SAA1*) expression was upregulated in muscle of critically ill patients. To test the relevance of these responses we assessed inflammation and acute-phase response at early and late time points in muscle of patients at risk for CIM.

**Design:**

Prospective observational clinical study and prospective animal trial.

**Setting:**

Two intensive care units (ICU) and research laboratory.

**Patients/Subjects:**

33 patients with Sequential Organ Failure Assessment scores ≥8 on 3 consecutive days within 5 days in ICU were investigated. A subgroup analysis of 12 patients with, and 18 patients without CIM (non-CIM) was performed. Two consecutive biopsies from *vastus lateralis* were obtained at median days 5 and 15, early and late time points. Controls were 5 healthy subjects undergoing elective orthopedic surgery. A septic mouse model and cultured myoblasts were used for mechanistic analyses.

**Measurements and Main Results:**

Early *SAA1* expression was significantly higher in skeletal muscle of CIM compared to non-CIM patients. Immunohistochemistry showed SAA1 accumulations in muscle of CIM patients at the early time point, which resolved later. *SAA1* expression was induced by IL-6 and tumor necrosis factor-alpha in human and mouse myocytes *in vitro*. Inflammation-induced muscular SAA1 accumulation was reproduced in a sepsis mouse model.

**Conclusions:**

Skeletal muscle contributes to general inflammation and acute-phase response in CIM patients. Muscular SAA1 could be important for CIM pathogenesis.

**Trial Registration:**

ISRCTN77569430.

## Introduction

Intensive care unit (ICU)-acquired weakness is a serious complication during critical illness, characterized by loss of muscle mass, preferential atrophy of fast-twitch myofibers and weakness [1]–[3]. The clinical consequences are prolonged hospital stay and mechanical ventilation, increased hospital mortality, and chronic physical disability [4], [5]. Diminished myosin heavy chain (MyHC) content is consistently observed [1], [6]. Others and we recently reported that non-excitable muscle membranes indicate patients at risk for critical illness myopathy (CIM) early during the disease process [1], [7], [8]. CIM deteriorates the disease course and leads to protracted rehabilitation, poor quality-of-life outcomes, and permanent disability [4], [5], [9]. The suffering and economic impact for the health care system and the society are high [10].

Earlier, we observed disturbed glucose utilization in skeletal muscle from CIM patients caused by insufficient translocation of the glucose transporter GLUT4 to the membrane [11]. Nonetheless, the pathophysiology of CIM is poorly understood [12]. General inflammation with sepsis, immobilization, sedation, hyperglycemia and corticosteroids contribute to CIM [13], [14]. Among these the mediators of inflammation interleukin 6 (IL-6) and tumor necrosis factor-alpha (TNF-α) are particularly important [15], [16]. Serum levels of IL-6 [14], [15] and TNF-α [16] are increased in systemic inflammatory response syndrome and sepsis patients and are associated with increased mortality [14]. Both IL-6 [17], [18] and TNF-α [19]–[21] contribute to muscular atrophy by increasing protein degradation [22], [23]. Both cytokines increase the expression of acute phase response proteins, such as serum amyloid A 1 (SAA1) in muscle and other tissues [24]. SAA1 is associated with muscle wasting and atrophy in cachectic mice [25]. In addition, IL-6 and SAA1 cooperate to enhance angiotensin (Ang) II-induced muscle atrophy [26]. However, it is unknown if inflammation induces acute phase response directly in myocytes of critically ill patients which contributes to CIM. We hypothesized that early identification of non-excitable muscle membranes indicative for CIM could be helpful to identify pathways involved in the pathogenesis of CIM. A gene expression array performed on skeletal muscle biopsies from CIM and non-CIM patients drew our attention to increased muscular *SAA1* gene expression indicative for acute phase response in muscle of CIM patients. We investigated factors regulating SAA1 synthesis in skeletal myocytes and tested conservation of this pathway in a sepsis mouse model.

## Materials and Methods

### Ethics Statement

The institutional review board of the Charité approved the study, and written informed consent was obtained from legal proxy (ICU patients), or the patients themselves (control subjects) (Charité EA2/061/06). The study was registered under http://www.controlled-trials.com, ISRCTN77569430. We specifically included patients at high risk to develop ICU-acquired weakness. Accordingly, critically ill, mechanically ventilated ICU-patients were eligible for inclusion once they showed Sequential Organ Failure Assessment (SOFA) scores ≥8 on three consecutive days within the first five days after ICU admission. In this observational study all patients (n = 33) were treated according to local standard operating procedures [27]. We have reported an analysis on defective glucose utilization in these same patients earlier [11]. All patients received physiotherapy by an experienced physiotherapist starting from day one in ICU. Passive range of motion and active exercise were prescribed daily based on interdisciplinary discussions involving physiotherapists, nurses, ward physicians and consultants, and according to individual patient needs. Study physicians assessed patients’ muscle strength according to the Medical Research Council (MRC) score. To be eligible for MRC score evaluation, patients had to be awake (defined as Richmond Agitation Sedation Scale scores of −1, 0, or +1) and to adequately respond to at least three out of five verbal commands as recently reported [8]. Five age- and gender-matched otherwise healthy patients undergoing elective orthopedic surgery permitted muscle biopsies.

Muscle membrane in-excitability after direct muscle stimulation is an accepted marker for early CIM diagnosis and was shown to be associated with later development of ICUAW [1], [8], [28]. We measured muscle membrane excitability after direct muscle stimulation at day 6 (4–13) in ICU as recently reported [1], [8]. Muscle membranes of CIM patients (n = 18) were non-excitable as shown by a reduced compound muscle action potential after direct muscle stimulation (dmCMAP<3 mV), whereas in non-CIM patients (n = 12) muscle membranes (CMAP≥3 mV) were excitable [8], [28], [29]. For logistical and clinical reasons three patients could not be classified and were consequently not included in comparisons. We took biopsy specimens from *vastus lateralis* muscle in all 33 ICU-patients at median day 5; referred to as early time point. Twenty-two patients were still in the ICU at median day 15, when a second biopsy was performed; referred to as late time point. Serum samples were taken at the second or third day on ICU and stored at −20°C.

Hematoxylin & Eosin and Gomori-trichrome histological staining on cryosections were performed to assess overall muscle pathology as described earlier [30]. Photographs were acquired with a Leica CTR 6500 microscope and a Leica DFC 360 FX digital camera. Further information about animal experiments, Microarray analyses, quantitative real-time RT-PCR (primer sequences are provided in Table S1), immunohistochemistry, ELISA, human and mouse myoblast culture and immunocytochemistry is provided in Methods S1.

### Statistical Tests

Non-parametric tests, the Mann-Whitney test to analyze group differences and the Wilcoxon test for dependent samples, were performed. Spearman’s rank correlation coefficients were calculated. Data are shown as median with interquartile range (IQR). Student’s t-tests and one-way ANOVA analyses were used for PCR data and cell culture experiments. Statistical tests were calculated using SPSS (version 19.0.0.1); box plots were made with the Sigma Plot software (version 12.0). Statistical significance was considered at *P*<0.05.

## Results

### A Gene Expression Array Analysis Uncovered Increased SAA1 Expression in CIM Muscle

**Figure 1:**
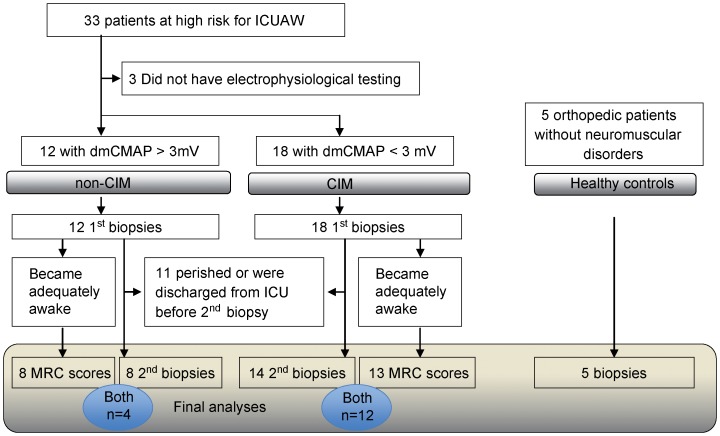
Study protocol.

The study design is outlined (Figure 1); data on patient characteristics and further clinical information are presented in Table 1. Patients with non-excitable muscle membrane indicating muscle pathology in CIM developed muscle weakness during ICU treatment, with a median MRC score of 3.0 (interquartile range [IQR], 2.9–3.3), whereas patients with excitable muscle membrane showed a median MRC score of 4.3 (IQR, 3.5–4.8; *P* = 0.003). A non-excitable muscle membrane measured at median day 6 (4/13) was predictive for the development of muscle weakness with a sensitivity and a specificity of 80% each.

**Table 1:** Characterization of critically ill patients.

Parameter	non-CIM-patients	CIM-patients
	dmCMAP≥3mV	dmCMAP<3mV
**N**	12	18
**Age** [years]	42.5 (32.5/57.5)	65.5 (41.0/76.0)
**Gender** [m/f]	7/5 (58.3%/41.7%)	15/3 83.3%/16.7%)
**BMI** [kg/m^2^]	26.0 (23.2/32.5)	27.9 (24.9/31.4)
**MRC Score**	4.3 (3.5/4.8)	3.0(2.9/3.3)†
**Diagnosis** [n (%)]		
ARDS	n = 3 (25.0%)	n = 8 (44.4%)
Trauma	n = 4 (33.3%)	n = 2 (11.1%)
Sepsis		n = 7 (38.9%)
CNS	n = 5 (41.7%)	n = 1 (5.6%)
**Time point of early biopsy** (days after ICU admission)	5.0 (4.0/6.5)	6.0 (4.0/7.0)
**Time point of late biopsy** (days after ICU admission)	15.0(14.0/16.0)	15.5(14.0/19.0)
**Survivors** n (%)	12 (100%)	12 (66.7%)
**Illness severity** at ICU admission		
SOFA	11.0 (9.0/14.0)	11.5 (10.0/14.0)
SAPS-II	53 (42/57)	62 (47/66)
**Treatment between ICU-admission and early biopsy**		
**Norepinephrine** [mg/d]	20.0 (8.8/21.7)	12.5 (9.0/24.3)
Patients with median **≥2 organ dysfunctions** until biopsied n (%)	2 (16.7%)	14 (77.8%)†
Patients with **acute renal failure** n (%)	1 (8.3%)	11 (61.1%)*
% of days with **septic shock**	8.3 (0.0/45.0)	45.0 (25.0/71.4)*

ICU indicates intensive care unit; BMI, body mass index; ARDS, acute respiratory distress syndrome; SOFA, Sequential Organ Failure Assessment; SAPS-II, Simplified Acute Physiology Score II; MRC, Medical Research Council; RASS, Richmond Agitation Sedation Scale. Results are expressed as medians with inter-quartile range or as absolute numbers with percentages. Differences are calculated between patients with excitable (non-CIM) and non-excitable (CIM) muscle membrane. Mann-Whitney test.

*P<0.05,

† *P*<0.01.

To assess very early changes in gene expression, we first performed microarrays. Among 24,133 transcripts, 1,841 genes were differentially expressed in critically ill patients *versus* control patients (5% FDR). The top 30 genes by fold-change increased or decreased in critical illness were revealing (Tables S2 and S3). Additionally, exon expression interaction with the grouping control *versus* CIM patients *versus* non-CIM patients led to a set of 1,948 significant differential expressed transcript clusters (5% FDR). Viewing the top 30 genes by fold change differences between CIM *versus* non-CIM patients implicated several candidates differentially regulated in CIM (Tables S4 and S5). Among these we found serum amyloid A 1 and 4 (*SAA1*, *SAA4*) and therefore reasoned that acute phase response occurred in muscle during critical illness, especially in CIM patients. The data discussed in this publication have been deposited in NCBI’s Gene Expression Omnibus and are accessible through GEO Series accession number GSE53702 (http://www.ncbi.nlm.nih.gov/geo/query/acc.cgi?acc=GSE53702).

**Figure 2:**
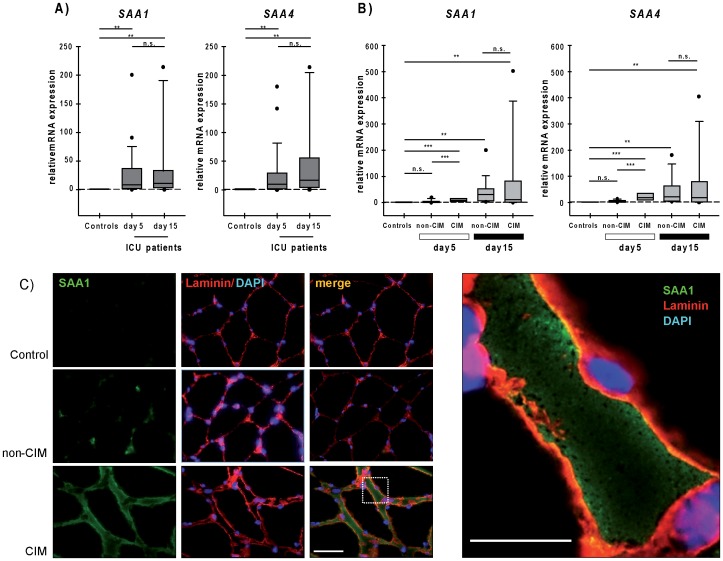
Muscular *SAA1* and *SAA4* expression and accumulation in CIM patients. Control values (no ICU subjects) were set to one and means were indicated as dashed lines. (A) RT-PCR analyses of *SAA1* and *SAA4* expression at early (day 5) and late (day 15) time points in *vastus lateralis* muscle of critically ill patients, and (B) CIM and non-CIM patients. *Glyceraldehyde-3 phosphate dehydrogenase* (*GAPDH*) expression was used as reference. Data are presented as box plots showing median, 25^th^ and 75^th^ percentiles. Wilcoxon tests were performed between early and late biopsy specimens and Mann-Whitney tests for the respective time points and controls. ***P<0.001, **P<0.01, *P<0.05, or n.s. (not statistically significant). (C) Immunohistochemistry of SAA1 (green) and the membrane marker laminin (red) on skeletal muscle biopsy specimens from control subjects, non-CIM and CIM patients. Nuclei were stained with 4′,6-diamidino-2-phenylindole (DAPI; blue); scale bar 50 μm. (D) Higher magnification of the merged picture from CIM patient in (C) to illustrate colocalization of SAA1 (green) and laminin (red) at the cell membrane, accumulation of SAA1 in the interstitium and around myofibers; scale bar 50 μm.

RT-PCR analyses on biopsy specimens confirmed the increased *SAA1* (controls: 0.09 (0.09–0.9), ICU-patients early time point: 8.16 (2.42–36.6), *P* = 0.005) and *SAA4* (controls: 0.55 (0.42–1.54), ICU-patients: 8.3 (2.56–29.39), *P* = 0.002) expression levels in critically ill patients. *SAA1* and *SAA4* expression remained unchanged between the early and late biopsy specimen (late time point: *SAA1∶*14.77 (4.13–27.2), *P* = 0.003; *SAA4∶*16.7 (6.18–40.64), *P* = 0.0478) (Figure 2A). Subgroup analyses showed that *SAA1* and *SAA4* were exclusively increased in CIM patients (*SAA1∶*30.6 (7.46–45.8), *P* = 0.001; *SAA4∶*22.8 (4.42–51.6), *P* = 0.0001), but remained unchanged in non-CIM (*SAA1∶*2.42 (0.53–4.6), *P* = 0.15; *SAA4∶*1.64 (1.06–6.64), *P* = 0.09) patients, in the early biopsy specimens (Figure 2B). *SAA1* and *SAA4* expression increased significantly in non-CIM patients (*SAA1∶*6.1 (4.99–15.1), *P* = 0.012; *SAA4∶*18.4 (8.47–36), *P* = 0.018), and reached the expression level of CIM patients (*SAA1∶*19.35 (1.34–67.2), *P* = 0.397 vs. early time point, *P* = 0.24 vs. non-CIM; *SAA4∶*16.71 (3.08–70.61), *P* = 0.06 vs. early time point, *P* = 0.916 vs. non-CIM) in the late biopsy specimens. In contrast, no further increase in *SAA1* and *SAA4* expression between the early and late biopsy specimens was observed in CIM patients (Figure 2B).

In addition to electrophysiological testing MRC scoring was possible in 21 out of the 30 ICU patients. In this subgroup, direct muscle stimulation identified weakness with a sensitivity of 80% and a specificity of 83.3%, which is consistent with our recent work [8] (Table S6). Based on MRC scoring (sum score <48 or mean MRC score <4) we performed a subgroup analysis and found an increased *SAA1* expression in patients with (n = 15) compared to patients without clinical evidence of weakness (n = 6) in the early biopsy specimens. *SAA4* expression was not different between both groups. Compared to controls *SAA1* and *SAA4* expression was increased in patients with clinical evidence of weakness at the early time point (Figure S1 and S2). Overall, these findings are consistent with the data shown here for electrophysiological classification of CIM (Figure 2B).

### SAA1 Production is Increased Early in Muscle of CIM Patients

**Figure 3:**
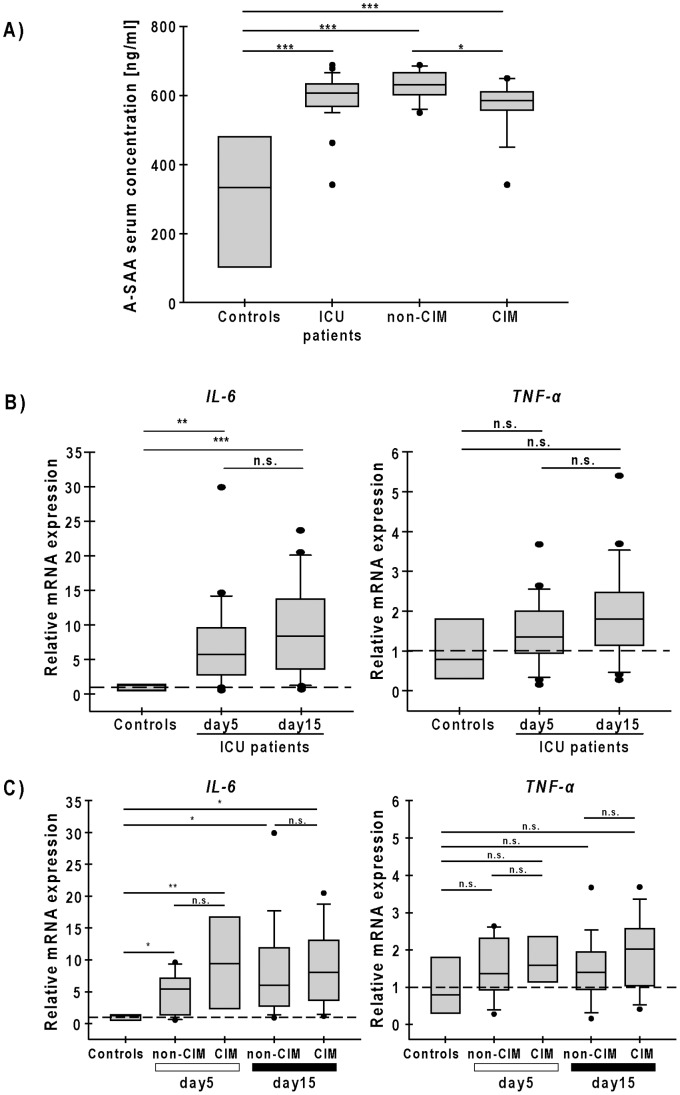
A-SAA serum levels and *IL-6* and *TNF-α* expression in skeletal muscle of critically ill patients. (A) Serum levels of acute phase SAA (A-SAA) measured by ELISA in healthy controls (n = 6), critically ill patients (ICUs, n = 30), non-CIM (n = 19) and CIM (n = 11) patients. Serum samples were obtained at days 2 to 3 after ICU admission. **P<0.01, *P<0.05. (B) RT-PCR analyses of *IL-6* and *TNF-α* expressions in skeletal muscle from critically ill patients at early (day 5) and late (day 15) time points. *Glyceraldehyde-3 phosphate dehydrogenase* (*GAPDH*) expression was used as reference. (C) RT-PCR analyses of *IL-6* and *TNF-α* expression at early and late time points in CIM and non-CIM patients. Data are presented as box plots showing median, 25^th^ and 75^th^ percentiles. Wilcoxon tests were performed between early and late biopsy specimens and Mann-Whitney tests for the respective time points and controls; ***P<0.001, **P<0.01, *P<0.05, or n.s. (not statistically significant).

We then proceeded to test our hypothesis that SAA1 production is induced in muscle of CIM but not non-CIM patients. Using immunohistochemistry SAA1 protein was found to accumulate in the interstitium, around myofibers, and at the sarcolemma were it co-localized with the membrane-marker laminin (Figure 2C and 2D). CIM patients showed stronger SAA1 accumulation in the early biopsy specimens, compared to non-CIM patients. The same differences, although diminished in SAA1 protein contents, were observed in the late biopsy specimens (Figure S3). These data suggested that high *SAA1* expression translated into a higher SAA1 protein content in skeletal muscle of CIM patients, and that SAA1 was directly synthesized by muscle. Acute phase SAA consist of both SAA1 and SAA2 [24]. Acute phase SAA is associated with generalized inflammation [24]. SAA serum levels were higher in ICU patients than in controls (controls: 333.7 (164.1–433.04), ICU-patients: 606.53 (570.95–631.53), *P*<0.01). However, SAA serum levels were not increased in CIM compared to non-CIM patients (CIM: 584.9 (560.45–610.34), non-CIM: 631.64 (609.63–650.4), *P*<0.05) (Figure 3A). These findings suggest that SAA synthesized in the skeletal muscle does not decisively contribute to circulating SAA levels.

Since IL-6 and TNF-α can increase SAA1 [31], we hypothesized that increased muscular IL-6 and/or TNF-α levels could be responsible for higher *SAA1* and *SAA4* expression in CIM patients. We determined *IL-6* and *TNF-α* mRNA expression in biopsy specimens of control and critically ill patients. *IL-6* expression (controls: 1.25 (0.51–1.38), ICU-patients early time point: 5.73 (2.4–9.51), *P*<0.0001), but not that of *TNF-α* (controls: 0.79 (0.34–1.45), ICU-patients early time point: 1.36 (0.96–1.9), *P* = 0.28) was elevated in critically ill patients (Figure 3B). Furthermore, no difference in *IL-6* and *TNF-α* expression was found between CIM and non-CIM patients (early time point: *IL-6:* CIM 6.02 (2.74–11.67), non-CIM 5.43 (2.45–7.11), *P* = 0.175; *TNF-α:* CIM 1.4 (0.97–1.84), non-CIM 1.31 (1.03–2.08), *P* = 0.76) or between the early and late biopsy specimens (late time point: *IL-6:* CIM 8.01 (4.324–10.37), non-CIM 8.92 (3.72–13.46); *TNF-α:* CIM 2.02 (1.15–2.55), non-CIM 1.58 (1.19–2.29), n.s.)(Figure 3B and 3C). These data suggest that inflammation occurred directly in muscle during critical illness.

**Figure 4:**
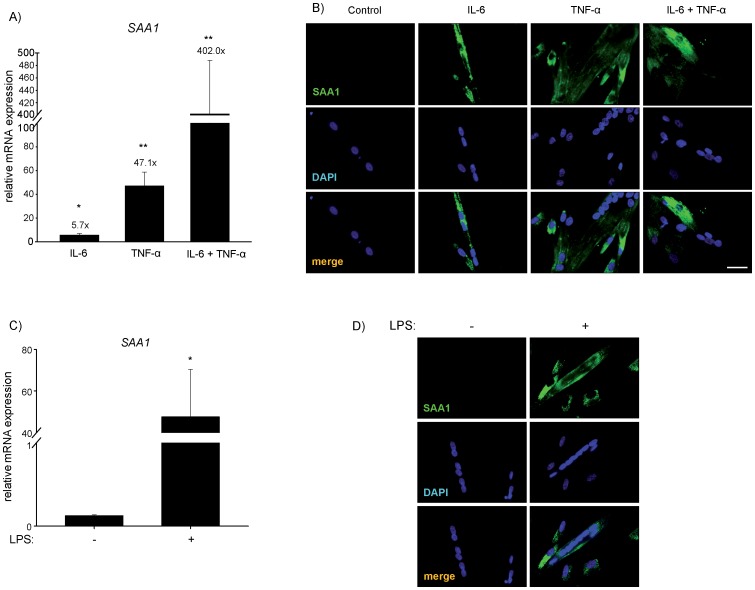
IL-6, TNF-α and LPS increased *SAA1* expression and protein content in human skeletal muscle cells *in vitro*. (A) Differentiated human skeletal myotubes were treated with human recombinant IL-6 (100 ng/ml), TNF-α (10 ng/ml), or a combination of both (IL-6, 100 ng/ml; TNF-α, 10 ng/ml) for 16 h. RT-PCR was used to measure *SAA1* expression, which was normalized to *beta-2-microglobulin* expression. Relative gene expression by fold-induction of *SAA1* expression (above column) is shown. **P<0.01, *P<0.05. (B) Immunocytochemistry of SAA1 (green) on differentiated human myotubes following treatment with human recombinant IL-6 (100 ng/ml), human recombinant TNF-α (10 ng/ml), and both cytokines (IL-6, 100 ng/ml; TNF-α, 10 ng/ml) together for 16 h is shown. Nuclei were stained with 4′,6-diamidino-2-phenylindole (DAPI; blue); scale bar 50 μm. (C) Human skeletal myotubes were treated with lipopolysaccharide (LPS, 1 μg/ml) for 16 h. RT-PCR was used to measure *SAA1* expression, which was normalized to *Glyceraldehyde-3 phosphate dehydrogenase* (*GAPDH*); *P<0.05. (D) Immunocytochemistry of SAA1 (green) on human myotubes following LPS treatment (1 μg/ml) for 16 h. Nuclei were stained with DAPI (blue); scale bar 50 μm.

Muscle biopsies consist of myocyte and non-myocyte cells, such as fibroblasts and endothelial cells. To test if myocytes synthesize SAA1, we performed cell culture experiments using human myocytes which were differentiated into myotubes. Indeed, using real-time RT-PCR we found *SAA1* to be endogenously expressed in myotubes. We treated myotubes with human recombinant IL-6, TNF-α, or both. Both IL-6 and TNF-α increased *SAA1* expression in human myotubes. A combination of IL-6 and TNF-α was more effective than either cytokine alone (Figure 4A). Immunofluorescence staining showed an increase in SAA1 protein (Figure 4B). Lipopolysaccharides (LPS) also mediate muscular atrophy [32]. LPS treatment of human myotubes increased *SAA1* expression (Figure 4C) and protein content (Figure 4D).

**Figure 5:**
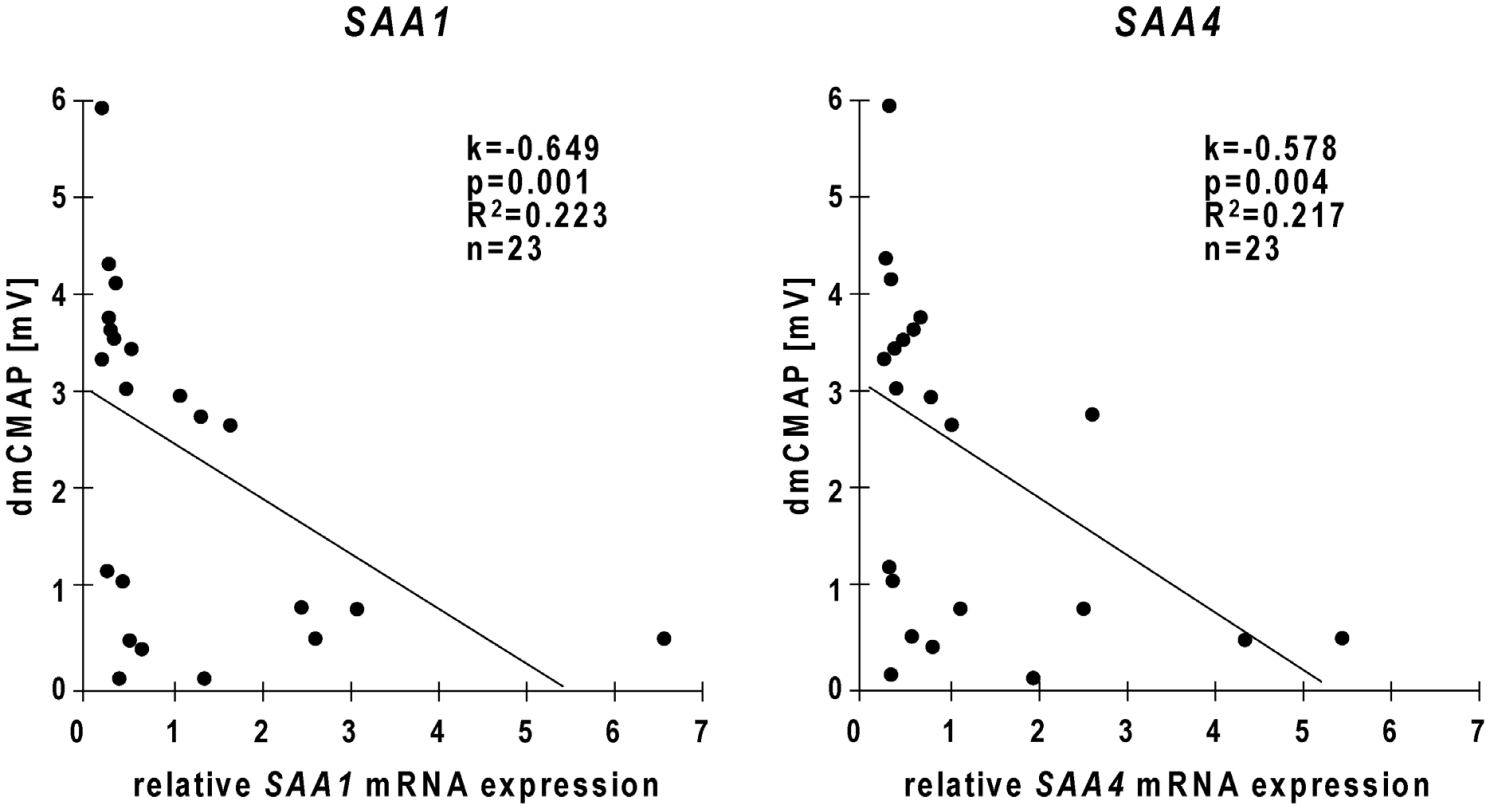
Early *SAA1* expression correlated with muscle membrane excitability of critically ill patients. A multivariate analysis was performed to test, if *SAA1* and *SAA4* expressions were correlated with clinical CIM parameters. *SAA1* (left) and *SAA4* (right) expressions measured in early biopsy specimens were inversely correlated with compound muscle action potential on direct muscle stimulation (dmCMAP).

To analyze if *SAA1* or *SAA4* expression were directly associated with compound muscle action potential, correlation analyses were performed. We found that *SAA1* and *SAA4* mRNA expression in the early biopsy specimen were inversely correlated with compound muscle action potential after direct muscle stimulation (dmCMAP) (Figure 5). In addition, *SAA1* and *SAA4* expression levels were directly correlated with each other (Figure S4).

### Inflammation-induced SAA1 Expression in Muscle is Conserved throughout Species

**Figure 6:**
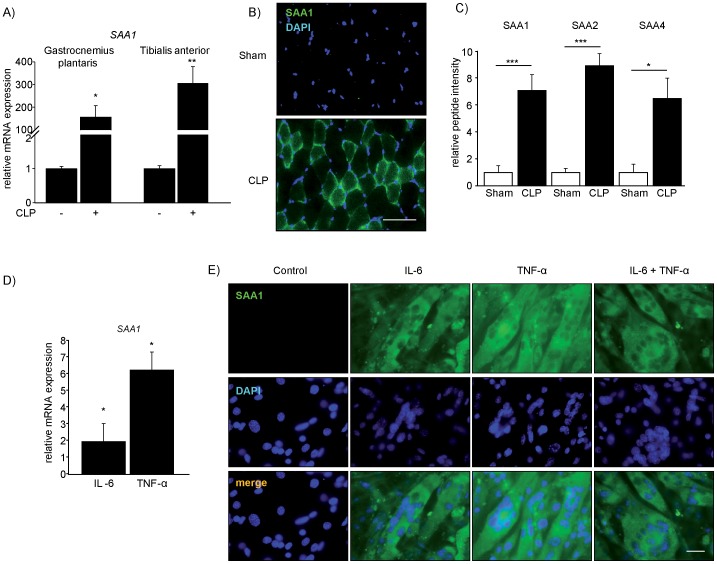
Sepsis and proinflammatory cytokines increase muscular SAA1 expression and protein content *in vivo* and *in vitro*. (A) Polymicrobial sepsis was induced by cecal ligation and puncture (CLP) in mice for 24 h (n = 5). Sham operated mice were used as controls (n = 5). RT-PCR was used to measure *SAA1* expression in *gastrocnemius plantaris* and *tibialis anterior* muscles, which was normalized to *GAPDH* expression. **P<0.01, *P<0.05. (B) Immunohistochemistry of SAA1 (green) on *gastrocnemius plantaris* muscle of sham and CLP treated mice (24 h treatment). Nuclei were stained with 4′,6-diamidino-2-phenylindole (DAPI; blue); scale bar 50 μm. (C) Mass-spectrometry was used to quantitate SAA1, SAA2 and SAA4 in dialysates of *vastus medialis* of sham (n = 8) and CLP (n = 8) 24 h after surgery. ***P<0.001, *P<0.05. (D) Differentiated mouse skeletal myotubes were treated with murine recombinant IL-6 (100 ng/ml) or murine recombinant TNF-α (10 ng/ml) for 16 h. RT-PCR was used to measure *SAA1* expression, which was normalized to *GAPDH*; *P<0.05. (E) Immunocytochemistry of SAA1 (green) on differentiated murine myotubes following treatment with murine recombinant IL-6 (100 ng/ml), murine recombinant TNF-α (10 ng/ml) or a combination of both (IL-6, 100 ng/ml; TNF-α, 10 ng/ml) for 16 h is shown. Nuclei were stained with DAPI (blue); scale bar 50 μm.

We next sought to test if inflammation-induced acute phase response in muscle is conserved throughout species. Therefore, we asked if our findings could be reproduced in a mouse model of polymicrobial sepsis. Wild type mice were subjected to the cecal ligation and puncture model (CLP) of sepsis, or a sham procedure, for 24 h [33]. RT-PCR analyses performed on the *gastrocnemius plantaris* and *tibialis anterior* confirmed increased muscular *SAA1* expression during sepsis (Figure 6A). Immunohistochemistry showed that SAA1 protein was increased in myofibers, at the sarcolemma around myofibers and in the interstitium of the *gastrocnemius plantaris* of septic mice (Figure 6B). To investigate if SAA protein was secreted by septic skeletal muscle and was contained in the muscular interstitium, we performed microdialysis in the *vastus medialis* of septic and sham mice 24 h after surgery. Mass-spectrometric analysis of dialysates showed an increase in interstitial SAA1, SAA2 and SAA4 proteins in *vastus medialis* of septic mice (Figure 6C). These data indicate that SAA is not only synthesized, but also secreted by skeletal muscle in response to inflammation. RT-PCR analyses and immunofluorescence stainings revealed that mouse myoblasts differentiated into myotubes also endogenously expressed SAA1. Differentiated myotubes were treated with murine recombinant IL-6 or TNF-α. Both IL-6 and TNF-α increased *SAA1* expression (Figure 6D). Immunofluorescence staining showed an increase in SAA1 protein in those myotubes (Figure 6E). LPS treatment of differentiated mouse myotubes increased *SAA1* expression and protein content (). These data indicate that inflammation-induced *SAA1* expression in myocytes is conserved throughout species.

## Discussion

We found that inflammation caused acute phase response in skeletal muscle of critically ill patients, which was associated with CIM. We demonstrated that early increases in *SAA1* and *SAA4* expression and SAA1 accumulation in muscle are associated with CIM development. *SAA1* was expressed in myocytes *in vitro*. Treatment with IL-6, TNF-α, or LPS increased its expression both in human and mouse skeletal muscle and myocytes. Inverse correlations between early *SAA1* expression and muscle membrane excitability suggest that SAA1 could contribute to the development of CIM.

Few data regarding differences in gene expression between CIM and non-CIM skeletal muscles are available. We relied on an expression array and identified genes that are specifically increased in CIM skeletal muscle at a very early time point after ICU admission. *SAA1* and *SAA4* expression was higher in skeletal muscle of CIM patients only in muscle biopsies of the early time point. The rapid and early increase in *SAA1* and *SAA4* expression could be responsible for SAA1 accumulation in the muscle membrane and interstitium of CIM patients, possibly overriding its degradation. SAA1 accumulation coincided with decreased electrical excitability of the muscle membrane measured at median day 6 in CIM patients. At the later time point, *SAA1* and *SAA4* expression were similar in CIM and non-CIM patients and SAA1 accumulation resolved; further supporting the hypothesis that early induction of SAA1 facilitates its accumulation in muscle membrane during inflammation. However, further experiments are needed to elucidate if these aggregates directly affect muscle membrane excitability especially during early critical illness. Our findings also implicate a very early pathomechanism facilitating CIM development right after ICU admission. This hypothesis is strengthened by the rapid increase in SAA1 synthesis and secretion in muscle of septic mice. Based on our findings, we hypothesize that skeletal muscle participates in acute phase responses that self-perpetuate muscle demise during sepsis.

The SAA family of proteins are sub-classified into acute phase response SAA1 and SAA2 [34], whose expression increases up to 1000-fold during inflammation [26], and SAA4, which is mainly constitutively expressed [35]. However, *SAA4* expression is also increased by inflammatory stimuli in various tissues [36]. Acute phase SAAs are predominantly synthesized and secreted by the liver in response to inflammation [24]. Clinical data suggest markedly increased inflammation in CIM patients [14]. We believe that this increase contributed to the early induction of *SAA1* and *SAA4* expression in muscle. However, for logistical reasons it was impossible to obtain biopsies earlier than five days after ICU admission. Therefore we took advantage of an animal model of polymicrobial sepsis enabling us to investigate much earlier time points of critical illness. With this model we showed that inflammation leads to a rapid increase of SAA1 synthesis in muscle. It also demonstrated that inflammation-induced acute phase response in muscle was conserved throughout species. Conservation of this pathway is also supported by our *in vitro* data showing increased SAA1 synthesis in response to cytokine treatment of murine myocytes. In addition, this mouse model proved to be useful to demonstrate increased SAA1 secretion in the muscular interstitium of septic mice.

The inflammatory cytokines IL-6, TNF-α, and IL-1β all increase hepatic SAA synthesis. However, skeletal muscle apparently also contributes to increased SAA production [26]. Although, *SAA* expression was higher in CIM than in non-CIM patients SAA serum levels were not increased in CIM patients. Possibly, SAA1 accumulations at the sarcolemma and in the interstitium might prevent SAA1’s entry into the circulation. Alternatively, SAA1 production in muscle is perhaps only a small fraction of the total SAA produced.

Both TNF-α and LPS increased *SAA1* expression *in vivo* [31], [37]. In line with these observations, our data showed that myocytes express *SAA1*, and that IL-6, TNF-α, and LPS all increase its basal expression. Since *IL-6* and *TNF-α* were equally expressed in CIM and non-CIM muscle, muscular IL-6 and TNF-α do not seem to be responsible for increased SAA1 or SAA4 contents in CIM. However, recently we reported that serum IL-6 levels were predictive of CIM [14]. We speculate that increased serum but not muscular IL-6 levels were responsible for increased *SAA1* expression in CIM muscle. A role for both SAA1 and IL-6 in muscular atrophy was recently reported [26]. SAA1 was found to be increased in skeletal muscle of cachectic mice with cancer [25]. In this work, *SAA1* expression correlated with the degree of skeletal muscle wasting and muscular atrophy. IL-6 and SAA1 were also shown to mediate skeletal muscle atrophy induced by AngII [26]. These data, and the findings we report here, implicate that SAA1 contributes to inflammation induced muscular atrophy.

Muscular *TNF-α* expression was unchanged in critically ill patients implicating a minor role of TNF-α in regulation of SAA1 expression. Nevertheless, we investigated the effect of TNF-α on SAA1 expression and protein content in myocytes *in vitro*. First, because *TNF-α* expression follows a time course during inflammation with an early increase and a rapid decrease after the inflammatory stimuli it is possible that the biopsy time point was too late to detect meaningful differences in *TNF-α* expression between CIM and non-CIM patients. Second, TNF-α increases *SAA1* expression in muscle and other tissues [24]. Third, we assumed that TNF-α [19]–[21] contributes to muscular atrophy in critically ill patients by increasing protein degradation [22], [23]. Fourth, it is unknown how much biological active TNF-α is contained in the skeletal muscle of our patients because TNF-α protein levels were not quantitated. Fifth, in general TNF-α serum levels [16] are increased in systemic inflammatory response syndrome and sepsis patients [14]. However, if TNF-α serum levels were elevated in our patients is uncertain.

We also found increased muscular *SAA4* expression in critically ill patients and higher upregulation in CIM muscle at the early time point. Indeed, SAA4 has been described as a minor acute phase reactant [36]. The positive correlation between *SAA1* and *SAA4* expression supports a possible common pathway regulating both genes during critical illness.

In these same patients, we recently reported that the glucose transporter GLUT4 a key regulator of glycemic homeostasis in skeletal muscle was trapped at perinuclear spaces of myocytes, most pronounced in patients with CIM, but resided at the sarcolemma in control subjects [11]. Glucose metabolism was not stimulated during euglycemic-hyperinsulinergic clamp. Interestingly, insulin signal transduction was intact and let to activation of Akt. In contrast, p-adenosine monophosphate-activated protein kinase (p-AMPK) was not detectable in CIM muscle. These observations [11] together with the measurement of non-excitable muscle membrane as well as membranous SAA1 accumulation reported here, all occurring early during CIM development, point towards a central role of the myocyte membrane in the pathogenesis of CIM. However, we have not yet identified the pathways that directly interconnect the metabolic disturbances we observed earlier and the inflammatory responses we report here in the same patients. But, avenues to do so certainly exist. For instance, the *SLC2A4* gene encoding GLUT4 is repressed by the inflammatory transcription factor NF-κB [38]. Thus, increased inflammation in CIM skeletal muscle could have let to an activation of the inflammation mediator NF-κB mediating downregulation of GLUT4. Finally, SAA is a known marker for insulin resistance [39].

We conclude that skeletal muscle contributes to general inflammation and acute-phase response in CIM patients. Differences in muscular SAA1 expression and content could be important for CIM pathogenesis.

### Limitations

We used muscle membrane excitability to differentiate between CIM and non-CIM patients. MRC scores could not be assessed for all patients mainly due to the fact that not all patients became awake during the study period. Therefore, the number of patients diagnosed with weakness based on MRC scoring is smaller compared to electrophysiological testing. Although, muscle membrane in-excitability after direct muscle stimulation is an accepted marker for early CIM diagnosis and correlates with ICUAW, membrane in-excitability and weakness are not synonymous [1], [8], [28]. However, direct muscle stimulation identified weakness with a sensitivity and specificity of 80% each, which is consistent with our recent work [8]. We conclude that electrophysiological testing is useful to predict weakness in patients who are not assessable by clinical measurements of muscle strength.

To get insights into early molecular changes in skeletal muscle caused by critical illness biopsies from the very beginning of the disease, preferably from day 1 if not hours after the onset of critical illness, are needed. However, according to German law and the ethic committee a legal proxy must give his or her informed consent before a muscle biopsy can be performed. Usually this process takes 3 to 5 days. At this time point molecular pathways leading to myopathy are already activated. Nevertheless, we identified early and specific changes in gene expression in the skeletal muscle of patients developing CIM. For the same reason, quantitation of *IL-6* and *TNF-α* expression might not be representative for the initial disease phase. Our findings that IL-6 and TNF-α increased SAA1 gene expression and protein content in human and mouse myotubes do not mean that they account for the observed changes in SAA1 expression and content between CIM and non-CIM patients; but we can also not exclude their involvement. The discrepancy between *IL-6* and *TNF-α* expression in human skeletal muscle, and the results of our cell culture and animal work in terms of SAA1 expression might be explained by differences in timing; early biopsies were performed at median day 5, animal experiments were performed after 24 hours of sepsis and cytokine treatment was performed for 16 hours *in vitro*. In addition, despite its association with CIM we did not prove that SAA1 is causatively linked to CIM development. Because we hypothesize that molecular changes in muscle of critically ill patients occur early during the disease process and based on the fact that it is difficult to obtain biopsy specimens much earlier than 3 days after onset of critical illness we think that cell culture and animal experiments are required to investigate mechanisms of inflammation induced muscle atrophy. Extrapolation of *in vivo* and *in vitro* data to the human situation needs to be done with caution.

## Supporting Information

Figure S1 **Muscular** ***SAA1*** **expression in patients with ICU-acquired weakness (ICUAW) according to dmCMAP or MRC scoring.** RT-PCR analyses of *SAA1* expression at the early time point in *vastus lateralis* muscle of critically ill patients with (A) excitable (n = 12) and non-excitable (n = 18) muscle membrane and (B) MRC score ≥ (n = 6) or <4 (n = 15) are shown. Control values (no ICU subjects) were set to one. *Glyceraldehyde-3 phosphate dehydrogenase* (*GAPDH*) expression was used as reference. Data are presented as box plots showing median, 25^th^ and 75^th^ percentiles.
(TIF)

Figure S2 **Muscular** ***SAA4*** **expression in patients with ICU-acquired weakness (ICUAW) according to dmCMAP or MRC scoring.** RT-PCR analyses of *SAA4* expression at the early time point in *vastus lateralis* muscle of critically ill patients with (A) excitable (n = 12) and non-excitable (n = 18) muscle membrane and (B) MRC score ≥ (n = 6) or <4 (n = 15) are shown. Control values (no ICU subjects) were set to one. *Glyceraldehyde-3 phosphate dehydrogenase* (*GAPDH*) expression was used as reference. Data are presented as box plots showing median, 25^th^ and 75^th^ percentiles.
(TIF)

Figure S3 **SAA1 accumulations were found in the skeletal muscle of CIM patients at the late time point.** Representative immunohistochemistry for SAA1 (green) and laminin (red) for the late time point of control subjects, CIM and non-CIM patients. Nuclei were stained with 4′,6-diamidino-2-phenylindole (DAPI; blue); scale bar 50 μm.
(TIF)

Figure S4 ***SAA1*** **and** ***SAA4*** **expression were positively correlated with each other.**
(TIF)

Figure S5 (A) Mouse skeletal myotubes were treated with lipopolysaccharide (LPS, 1 μg/ml) for 16 h. RT-PCR was used to measure *SAA1* expression, which was normalized to *Glyceraldehyde-3 phosphate dehydrogenase* (*Gapdh*); *P<0.05. (B) Immunocytochemistry of SAA1 (green) on murine myotubes following LPS treatment (1 μg/ml) for 16 h. Nuclei were stained with 4′,6-diamidino-2-phenylindole (DAPI; blue); scale bar 50 μm.
(TIF)

Table S1 **Primer pairs for RT-PCR are shown.** SAA indicates serum amyloid A; GAPDH, glyceraldehyde-3-phosphate dehydrogenase; Hs, Homo sapiens; Mm, Mouse musculus.
(DOC)

Table S2 Top 30 genes increased in vastus lateralis of ICU patients.
(DOC)

Table S3 Top 30 genes decreased in vastus lateralis of ICU patients.
(DOC)

Table S4 Top 30 genes increased in vastus lateralis of CIM compared to non-CIM patients.
(DOC)

Table S5 Top 30 genes decreased in vastus lateralis of CIM compared to non-CIM patients.
(DOC)

Table S6 **Direct muscle stimulation identified weakness with a sensitivity of 80% and a specificity of 83.3%.**
(DOC)

Methods S1 **Details about electrophysiological measurements, muscle biopsies, microarray analyzes and quantitative real-time PCR, the animal model of polymicrobial sepsis by cecal ligation and puncture surgery, mass spectrometry, immunohistochemistry and ELISA, and human and murine myoblast culture, RT-PCR, and immunofluorescence are provided.**
(DOC)
