# Supplementary figures and images for: Inflammation-Induced Acute Phase Response in Skeletal Muscle and Critical Illness Myopathy
Source: PLoS One. 2014 Mar 20;9(3):e92048. doi: 10.1371/journal.pone.0092048 (PMC3961297; doi:10.1371/journal.pone.0092048)

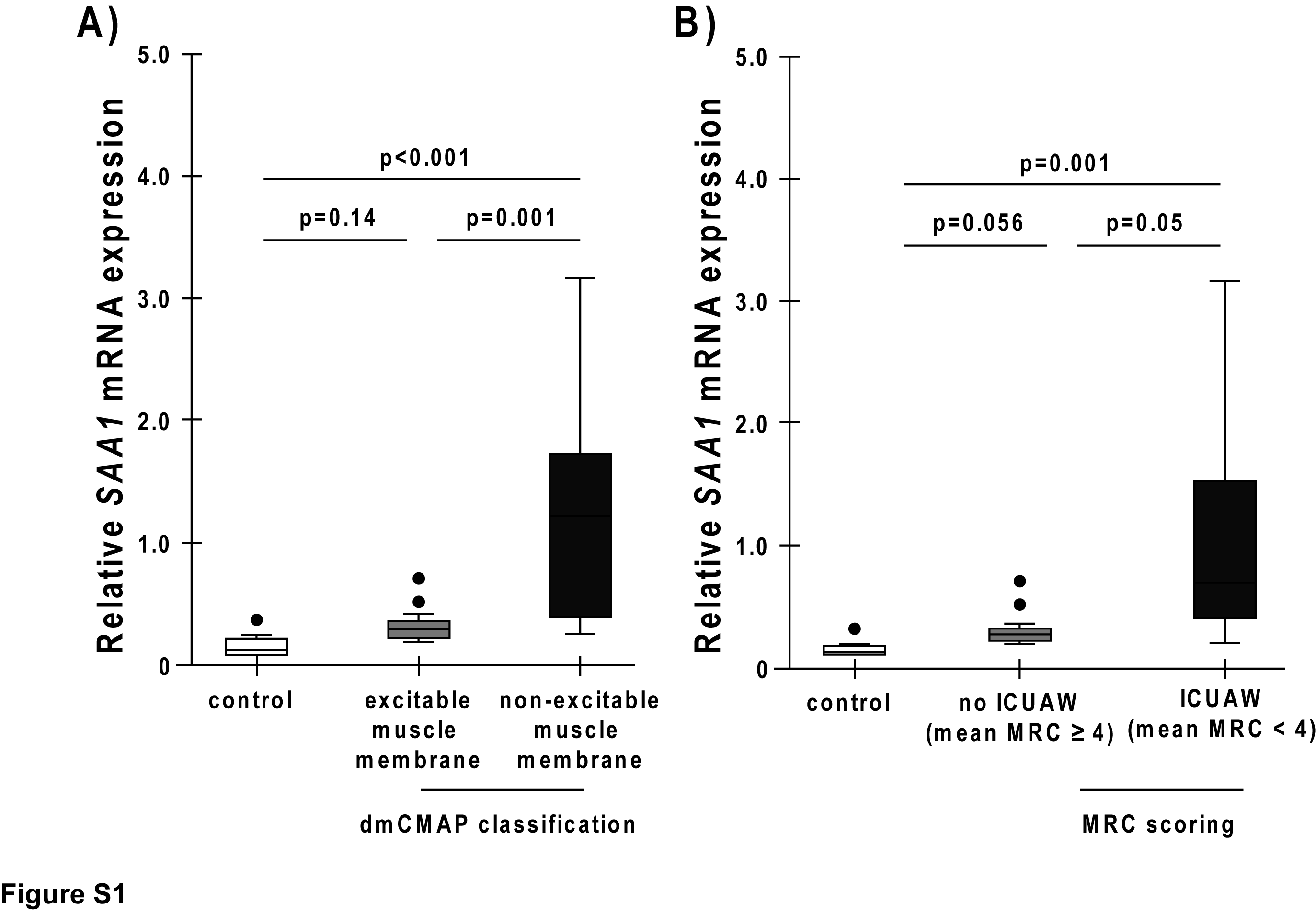

Supplement: Figure S1 — Muscular SAA1 expression in patients with ICU-acquired weakness (ICUAW) according to dmCMAP or MRC scoring. RT-PCR analyses of SAA1 expression at the early time point in vastus lateralis muscle of critically ill patients with (A) excitable (n = 12) and non-excitable (n = 18) muscle membrane and (B) MRC score ≥ (n = 6) or <4 (n = 15) are shown. Control values (no ICU subjects) were set to one. Glyceraldehyde-3 phosphate dehydrogenase (GAPDH) expression was used as reference. Data are presented as box plots showing median, 25th and 75th percentiles. (TIF) [file pone.0092048.s001.tif]

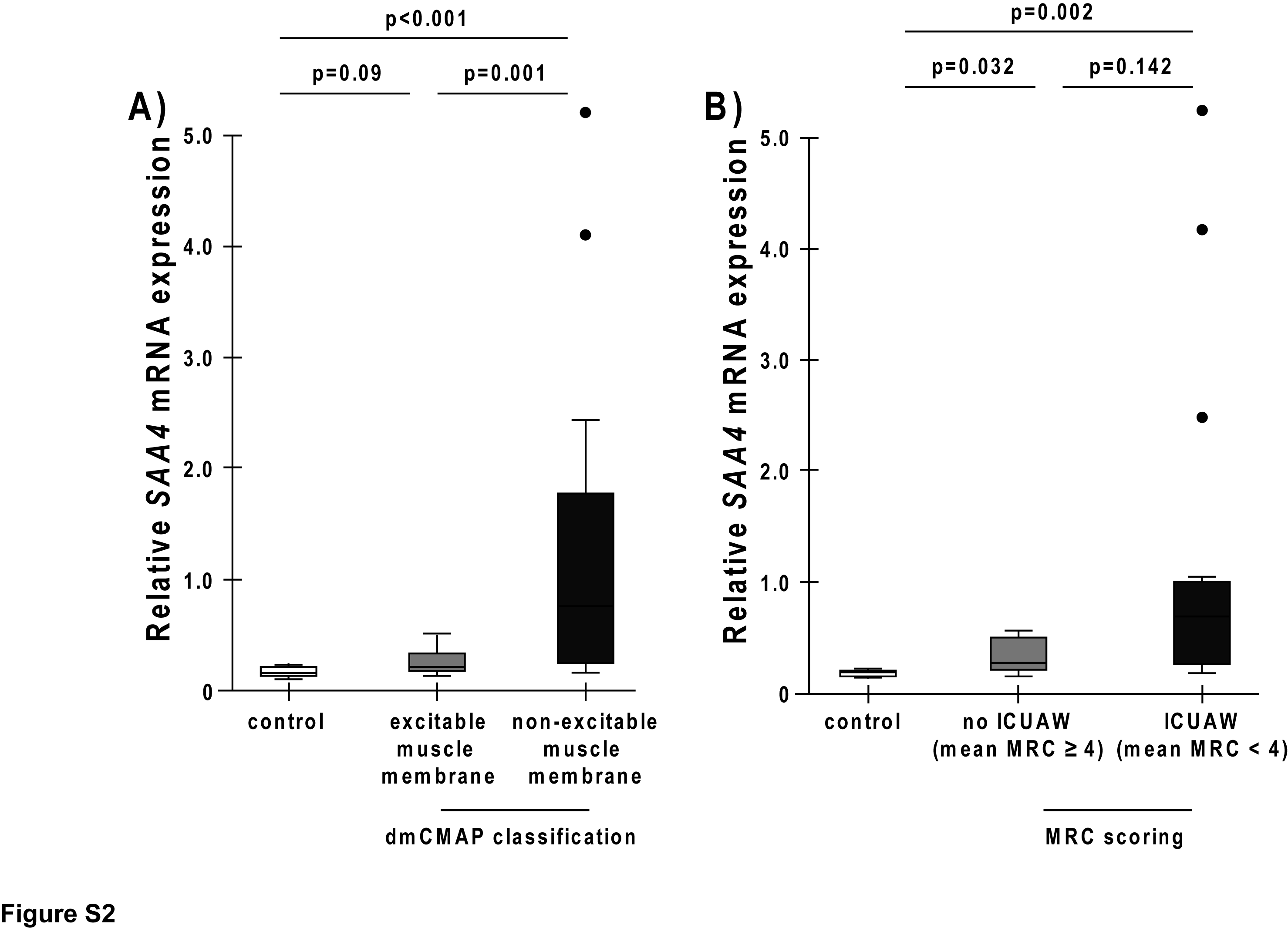

Supplement: Figure S2 — Muscular SAA4 expression in patients with ICU-acquired weakness (ICUAW) according to dmCMAP or MRC scoring. RT-PCR analyses of SAA4 expression at the early time point in vastus lateralis muscle of critically ill patients with (A) excitable (n = 12) and non-excitable (n = 18) muscle membrane and (B) MRC score ≥ (n = 6) or <4 (n = 15) are shown. Control values (no ICU subjects) were set to one. Glyceraldehyde-3 phosphate dehydrogenase (GAPDH) expression was used as reference. Data are presented as box plots showing median, 25th and 75th percentiles. (TIF) [file pone.0092048.s002.tif]

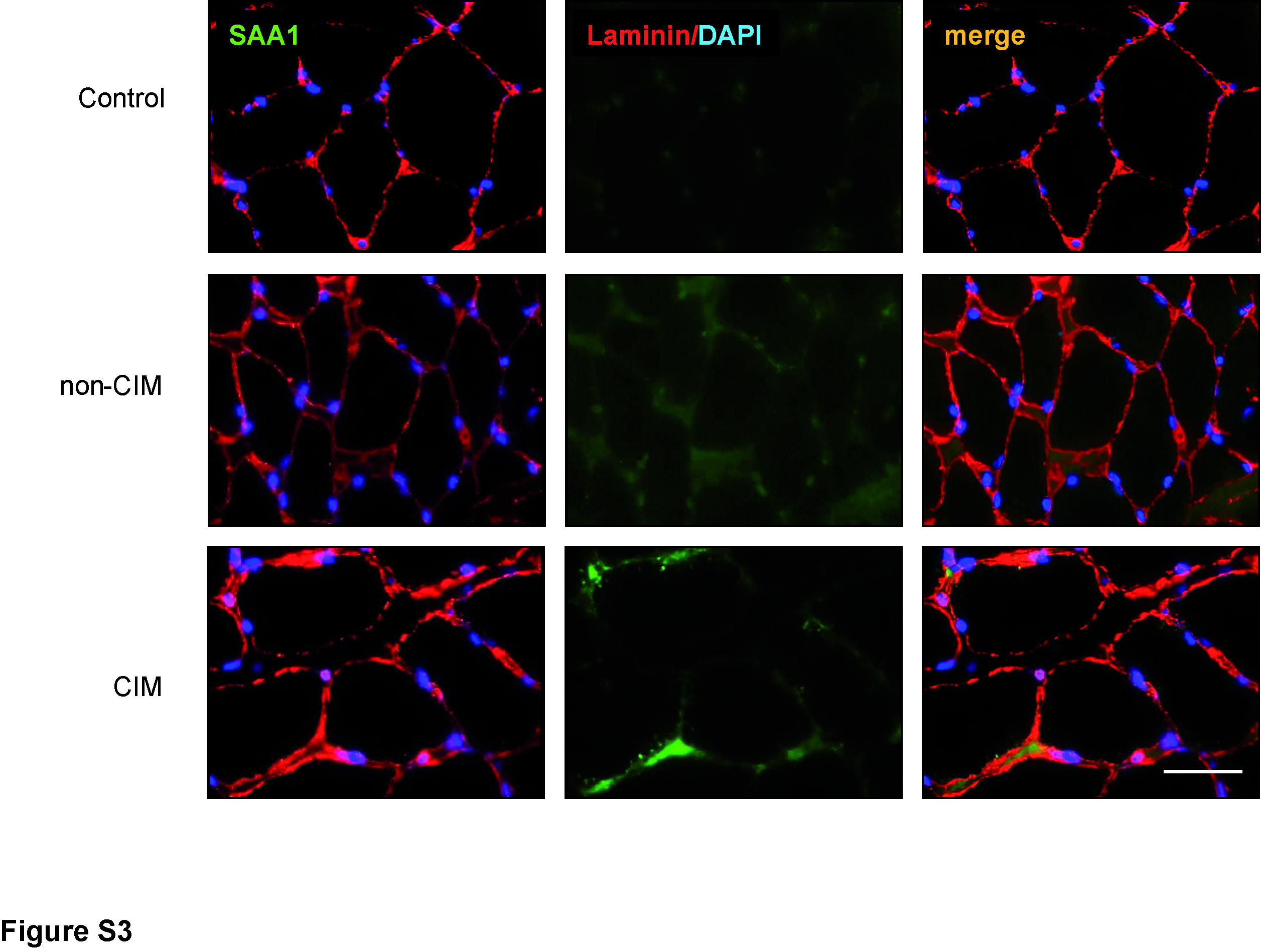

Supplement: Figure S3 — SAA1 accumulations were found in the skeletal muscle of CIM patients at the late time point. Representative immunohistochemistry for SAA1 (green) and laminin (red) for the late time point of control subjects, CIM and non-CIM patients. Nuclei were stained with 4′,6-diamidino-2-phenylindole (DAPI; blue); scale bar 50 μm. (TIF) [file pone.0092048.s003.tif]

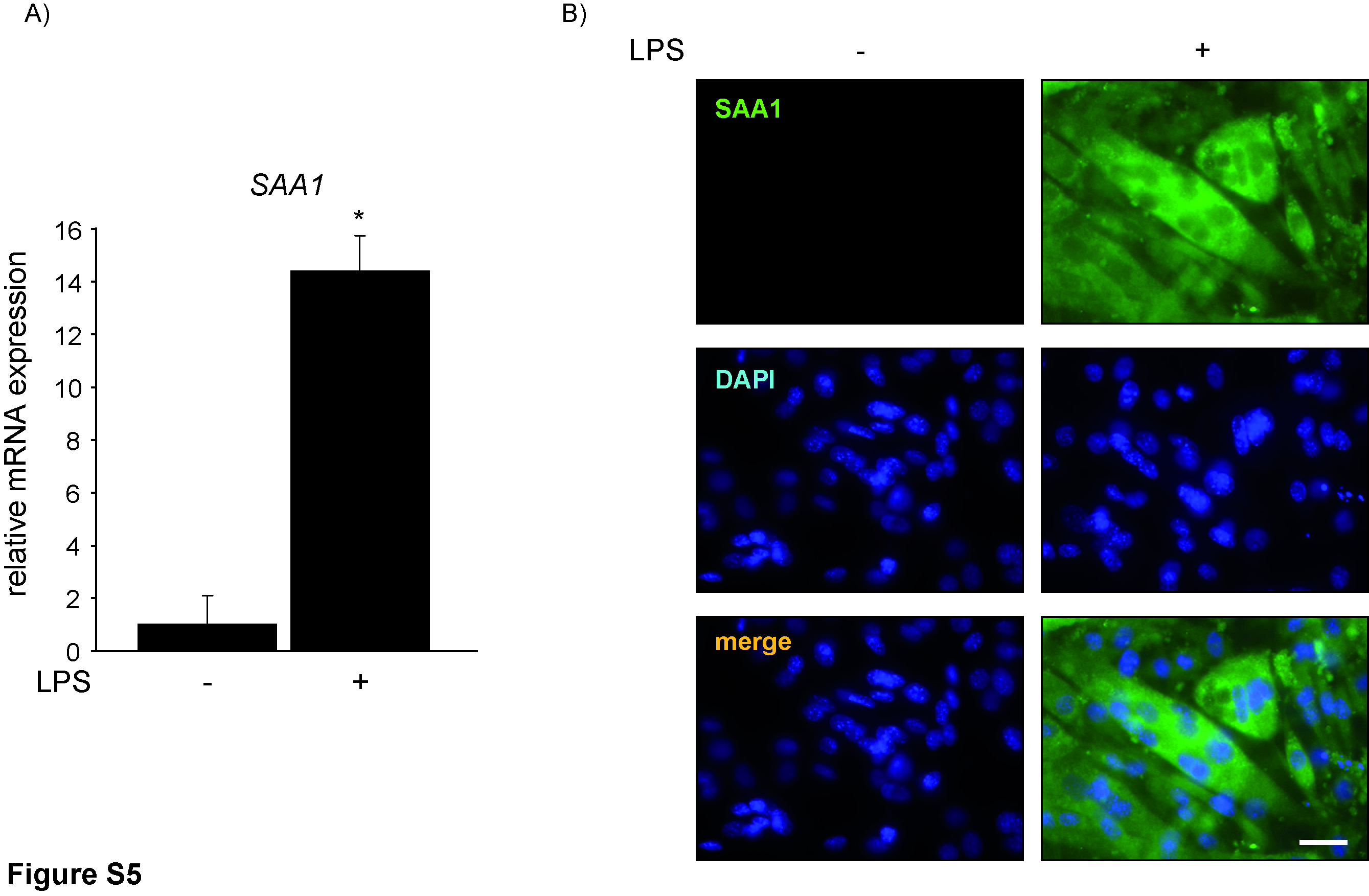

Supplement: Figure S5 — (A) Mouse skeletal myotubes were treated with lipopolysaccharide (LPS, 1 μg/ml) for 16 h. RT-PCR was used to measure SAA1 expression, which was normalized to Glyceraldehyde-3 phosphate dehydrogenase (Gapdh); *P<0.05. (B) Immunocytochemistry of SAA1 (green) on murine myotubes following LPS treatment (1 μg/ml) for 16 h. Nuclei were stained with 4′,6-diamidino-2-phenylindole (DAPI; blue); scale bar 50 μm. (TIF) [file pone.0092048.s005.tif]
