# Supplementary material for: Inflammation-Induced Acute Phase Response in Skeletal Muscle and Critical Illness Myopathy
Source: PLoS One. 2014 Mar 20;9(3):e92048. doi: 10.1371/journal.pone.0092048 (PMC3961297; doi:10.1371/journal.pone.0092048)
Supplement: Table S1 — Primer pairs for RT-PCR are shown. SAA indicates serum amyloid A; GAPDH, glyceraldehyde-3-phosphate dehydrogenase; Hs, Homo sapiens; Mm, Mouse musculus. (DOC) [file pone.0092048.s006.doc]

**Table S1**

*Primer pairs for RT-PCR are shown.*

| **Primer name** | **Primer sequence** |
| --- | --- |
| Hs_SAA1_forward | 5’-TGG TTT TCT GCT CCT TGG TC-3’ |
| Hs_SAA1_reverse | 5’-AGA GTA GGC TCT CCA CAT GTC C-3’ |
| Mm_SAA1_forward | 5’- CCA GGA TGA AGC TAC TCA CCA -3’ |
| Mm_SAA1_reverse | 5’- TAG GCT CGC CAC ATG TCC -3’ |
| Hs_SAA4_forward | 5’-CCA GCA GCT CTG CCT TTA CT-3’ |
| Hs_SAA4_reverse | 5’-TGT GAA AAG CCT CAT TGT GC-3’ |
| Hs_beta-2-Microglobulin_forward | 5’-CTC TCT TTC TGG CCT GGA GG-3’ |
| Hs_beta-2-Microglobulin_reverse | 5’-TGC TGG ATG ACG TGA GTA AAC C-3’ |
| Hs_GAPDH_forward | 5’-AGC CAC ATC GCT CAG ACA C-3’ |
| Hs_GAPDH_reverse | 5’-GCC CAA TAC GAC CAA ATC C-3’ |
| Mm_Gapdh_forward | 5’- ATG GTG AAG GTC CGT GTG A -3’ |
| Mm_Gapdh_reverse | 5’- AAT CTC CAC TTT GCC ACT GC -3’ |
