# Supplementary material for: Inflammation-Induced Acute Phase Response in Skeletal Muscle and Critical Illness Myopathy
Source: PLoS One. 2014 Mar 20;9(3):e92048. doi: 10.1371/journal.pone.0092048 (PMC3961297; doi:10.1371/journal.pone.0092048)
Supplement: Table S2 — Top 30 genes increased in vastus lateralis of ICU patients. (DOC) [file pone.0092048.s007.doc]

**Table S2**

***Top 30 genes increased in vastus lateralis*** of ICU patients.

| **Probe Set** | **Symbol** | **RefSeq ID** | **Fold-Change** | **p-value** | **FDR** |
| --- | --- | --- | --- | --- | --- |
| 3907190 | SLPI | NM_003064 | 15.07 | 1.19E-04 | 1.12E-02 |
| 2532378 | CHRND | NM_000751 | 11.55 | 8.38E-04 | 2.37E-02 |
| 2587937 | CHRNA1 | NM_001039523 | 11.46 | 4.50E-06 | 3.88E-03 |
| 3358241 | SCT | NM_021920 | 10.97 | 3.01E-05 | 7.12E-03 |
| 2341083 | GADD45A | NM_001924 | 10.40 | 6.40E-04 | 2.12E-02 |
| 3779314 | CHMP1B | NM_020412 | 9.96 | 5.09E-06 | 4.20E-03 |
| 2394478 | CHD5 | NM_015557 | 9.53 | 3.77E-03 | 4.97E-02 |
| 3662106 | MT1A | NM_005946 | 8.27 | 1.54E-03 | 3.15E-02 |
| 3567187 | DHRS7 | NM_016029 | 8.09 | 1.12E-07 | 1.66E-03 |
| 3862273 | PRR13 | NM_018457 | 7.83 | 4.62E-04 | 1.85E-02 |
| 3662086 | MT4 | NM_032935 | 7.73 | 4.48E-04 | 1.83E-02 |
| 2340961 | IL12RB2 | NM_001559 | 7.05 | 8.10E-07 | 1.84E-03 |
| 3014291 | FLJ30064 | AK054626 | 5.83 | 1.94E-03 | 3.54E-02 |
| 4023901 | LOC158696 | NR_026935 | 5.74 | 3.42E-04 | 1.68E-02 |
| 3930360 | RUNX1 | NM_001001890 | 5.66 | 7.67E-04 | 2.30E-02 |
| 2339740 | EFCAB7 | NM_032437 | 5.64 | 3.24E-04 | 1.65E-02 |
| 2822492 | C5orf30 | BC009203 | 5.61 | 3.00E-04 | 1.62E-02 |
| 3321150 | ARNTL | NM_001178 | 5.38 | 1.80E-03 | 3.39E-02 |
| 2610359 | IRAK2 | NM_001570 | 5.06 | 5.17E-04 | 1.91E-02 |
| 2360257 | IL6R | NM_000565 | 5.06 | 2.67E-03 | 4.13E-02 |
| 3594892 | FLJ27352 | AK130862 | 5.05 | 2.56E-06 | 2.94E-03 |
| 2436826 | KCNN3 | NM_002249 | 4.98 | 6.24E-04 | 2.09E-02 |
| 3303392 | BLOC1S2 | NM_001001342 | 4.97 | 6.67E-05 | 9.10E-03 |
| 3453533 | DDN | NM_015086 | 4.97 | 2.32E-03 | 3.86E-02 |
| 3572982 | POMT2 | NM_013382 | 4.85 | 4.45E-05 | 8.07E-03 |
| 3064024 | C7orf61 | BC031966 | 4.85 | 2.45E-05 | 6.59E-03 |
| 3977347 | CCNB3 | NM_033031 | 4.82 | 1.05E-03 | 2.61E-02 |
| 3135929 | SBF1P1 | NR_027765 | 4.82 | 3.43E-04 | 1.68E-02 |
| 3258477 | PLCE1 | NM_016341 | 4.72 | 9.49E-05 | 1.00E-02 |
| 3025740 | TMEM140 | NM_018295 | 4.66 | 2.19E-04 | 1.43E-02 |
