# Supplementary material for: Inflammation-Induced Acute Phase Response in Skeletal Muscle and Critical Illness Myopathy
Source: PLoS One. 2014 Mar 20;9(3):e92048. doi: 10.1371/journal.pone.0092048 (PMC3961297; doi:10.1371/journal.pone.0092048)
Supplement: Table S3 — Top 30 genes decreased in vastus lateralis of ICU patients. (DOC) [file pone.0092048.s008.doc]

**Table S3**

***Top 30 genes decreased in vastus lateralis*** of ICU patients.

| **Probe Set** | **Symbol** | **RefSeq ID** | **Fold-Change** | **p-value** | **FDR** |
| --- | --- | --- | --- | --- | --- |
| 2830450 | NPY6R | NR_002713 | -29.53 | 2.07E-05 | 6.59E-03 |
| 2712932 | C3orf43 | NM_001077657 | -26.88 | 1.27E-06 | 2.31E-03 |
| 3802396 | AQP4 | NM_001650 | -24.54 | 4.40E-05 | 8.05E-03 |
| 3429857 | C12orf75 | NM_001145199 | -17.20 | 1.65E-05 | 6.18E-03 |
| 3431731 | LOC100131138 | NR_036513 | -16.67 | 1.48E-06 | 2.35E-03 |
| 2672442 | MYL3 | NM_000258 | -13.70 | 1.24E-05 | 5.52E-03 |
| 3480508 | IL17D | NM_138284 | -13.09 | 1.26E-06 | 2.31E-03 |
| 2622607 | SLC38A3 | NM_006841 | -12.65 | 3.89E-07 | 1.66E-03 |
| 3288626 | LOC100509279 | XR_114364 | -12.14 | 2.37E-03 | 3.92E-02 |
| 3689979 | MYLK3 | NM_182493 | -11.96 | 7.93E-04 | 2.34E-02 |
| 3744919 | DHRS7C | NM_001105571 | -11.71 | 2.82E-06 | 2.95E-03 |
| 3689971 | MYLK3 | NM_182493 | -11.23 | 6.43E-05 | 9.02E-03 |
| 3499682 | LOC121952 | NR_026965 | -11.12 | 7.32E-05 | 9.19E-03 |
| 2709631 | MASP1 | NM_001879 | -10.14 | 7.18E-07 | 1.84E-03 |
| 3720383 | TCAP | NM_003673 | -9.99 | 1.12E-03 | 2.68E-02 |
| 3689973 | MYLK3 | NM_182493 | -9.92 | 1.21E-03 | 2.81E-02 |
| 3689981 | MYLK3 | NM_182493 | -9.84 | 2.60E-04 | 1.49E-02 |
| 3866821 | BSPH1 | NM_001128326 | -9.55 | 3.54E-04 | 1.70E-02 |
| 4013157 | LDHB | NM_002300 | -9.48 | 8.32E-04 | 2.37E-02 |
| 2697372 | TXNDC6 | NM_178130 | -9.18 | 3.18E-04 | 1.64E-02 |
| 3215560 | FBP2 | NM_003837 | -9.13 | 7.75E-04 | 2.31E-02 |
| 2530183 | LOC646736 | BC017935 | -9.08 | 3.76E-04 | 1.73E-02 |
| 3870758 | LILRA5 | NM_021250 | -9.08 | 2.38E-04 | 1.47E-02 |
| 2583465 | ITGB6 | NM_000888 | -8.89 | 1.40E-03 | 3.03E-02 |
| 3443226 | MFAP5 | NM_003480 | -8.78 | 1.31E-03 | 2.94E-02 |
| 2672376 | PRSS42 | NM_182702 | -8.72 | 1.74E-05 | 6.18E-03 |
| 2983738 | DKFZp451B082 | NR_033862 | -8.63 | 6.59E-07 | 1.84E-03 |
| 3258713 | LGI1 | NM_005097 | -8.53 | 8.85E-05 | 9.76E-03 |
| 3563687 | C14orf138 | NM_024558 | -8.44 | 6.80E-05 | 9.10E-03 |
| 2443120 | DPT | NM_001937 | -8.25 | 7.17E-06 | 4.43E-03 |
