# Supplementary material for: Inflammation-Induced Acute Phase Response in Skeletal Muscle and Critical Illness Myopathy
Source: PLoS One. 2014 Mar 20;9(3):e92048. doi: 10.1371/journal.pone.0092048 (PMC3961297; doi:10.1371/journal.pone.0092048)
Supplement: Table S4 — Top 30 genes increased in vastus lateralis of CIM compared to non-CIM patients. (DOC) [file pone.0092048.s009.doc]

**Table S4**

***Top 30 genes increased in vastus lateralis of CIM compared to non-***CIM patients.

| **Probe Set** | **Symbol** | **RefSeq ID** | **Fold-Change** | **FDR** | **LSD p-value** |
| --- | --- | --- | --- | --- | --- |
| 2676397 | ITIH4 | NM_002218 | 6.79 | 3.27E-03 | 1.41E-02 |
| 3365318 | SAA4 | NM_006512 | 6.58 | 7.83E-04 | 2.10E-02 |
| 3486096 | FREM2 | NM_207361 | 5.30 | 1.18E-02 | 3.55E-03 |
| 2471384 | KCNS3 | NM_002252 | 5.15 | 2.95E-02 | 1.35E-03 |
| 3989110 | ATP1B4 | NM_001142447 | 4.58 | 1.63E-05 | 1.77E-03 |
| 2353988 | FAM46C | NM_017709 | 4.03 | 8.93E-04 | 8.91E-03 |
| 3800898 | MIR1-2 | NR_029662 | 4.02 | 1.92E-02 | 4.30E-02 |
| 3407849 | C12orf39 | NM_030572 | 4.00 | 2.76E-02 | 4.75E-02 |
| 3322700 | SAA1 | NM_000331 | 3.99 | 8.35E-03 | 2.80E-02 |
| 3913335 | C20orf166 | NM_178463 | 3.97 | 4.81E-04 | 8.39E-03 |
| 2401670 | MYOM3 | NM_152372 | 3.97 | 2.26E-07 | 1.73E-02 |
| 2663927 | GRIP2 | NM_001080423 | 3.74 | 2.24E-10 | 9.44E-04 |
| 2374926 | SHISA4 | NM_198149 | 3.34 | 4.78E-02 | 4.33E-02 |
| 3294159 | P4HA1 | NM_000917 | 3.18 | 1.39E-02 | 3.77E-03 |
| 2643592 | EPHB1 | NM_004441 | 3.01 | 1.37E-03 | 1.53E-02 |
| 2880292 | DPYSL3 | NM_001197294 | 2.97 | 7.74E-03 | 2.10E-02 |
| 3118818 | PTP4A3 | NM_032611 | 2.96 | 2.84E-03 | 1.84E-03 |
| 3302693 | LOXL4 | NM_032211 | 2.87 | 8.59E-03 | 4.15E-03 |
| 3758845 | HDAC5 | NM_005474 | 2.84 | 5.59E-04 | 2.16E-04 |
| 2676167 | TNNC1 | NM_003280 | 2.83 | 1.03E-05 | 1.47E-05 |
| 3526831 | RASA3 | NM_007368 | 2.74 | 3.20E-02 | 9.80E-03 |
| 2494709 | CNNM4 | NM_020184 | 2.66 | 5.70E-08 | 9.83E-04 |
| 2924851 | RSPO3 | NM_032784 | 2.56 | 9.40E-06 | 4.04E-02 |
| 2429556 | CASQ2 | NM_001232 | 2.54 | 2.99E-03 | 5.72E-03 |
| 3294668 | SYNPO2L | NM_001114133 | 2.52 | 2.81E-05 | 1.31E-02 |
| 3893458 | PPDPF | NM_024299 | 2.41 | 7.49E-04 | 3.60E-02 |
| 3726569 | SPATA20 | NM_022827 | 2.40 | 1.03E-02 | 2.71E-02 |
| 3453882 | MCRS1 | NM_006337 | 2.39 | 9.57E-04 | 1.15E-02 |
| 2712932 | C3orf43 | NM_001077657 | 2.38 | 7.76E-17 | 3.09E-02 |
| 2881300 | CAMK2A | NM_015981 | 2.34 | 2.73E-03 | 3.08E-03 |
