# Supplementary material for: Inflammation-Induced Acute Phase Response in Skeletal Muscle and Critical Illness Myopathy
Source: PLoS One. 2014 Mar 20;9(3):e92048. doi: 10.1371/journal.pone.0092048 (PMC3961297; doi:10.1371/journal.pone.0092048)
Supplement: Table S5 — Top 30 genes decreased in vastus lateralis of CIM compared to non-CIM patients. (DOC) [file pone.0092048.s010.doc]

**Table S5**

***Top 30 genes decreased in vastus lateralis of CIM compared to non-***CIM patients.

| **Probe Set** | **Symbol** | **RefSeq ID** | **Fold-Change** | **FDR** | **LSD p-value** |
| --- | --- | --- | --- | --- | --- |
| 3452417 | SLC38A4 | NM_018018 | -4.65 | 2.73E-02 | 1.81E-02 |
| 3321150 | ARNTL | NM_001178 | -3.67 | 1.31E-21 | 5.18E-03 |
| 2341083 | GADD45A | NM_001924 | -3.56 | 1.05E-05 | 3.61E-02 |
| 3233605 | PFKFB3 | NM_004566 | -3.55 | 1.61E-13 | 3.65E-02 |
| 2709132 | ETV5 | NM_004454 | -3.35 | 2.37E-02 | 8.98E-03 |
| 2923939 | SMPDL3A | NM_006714 | -3.18 | 7.99E-04 | 3.75E-03 |
| 2548617 | CDC42EP3 | NM_006449 | -2.95 | 1.31E-02 | 1.06E-02 |
| 3732885 | PRKAR1A | NM_212472 | -2.91 | 5.11E-05 | 4.98E-03 |
| 2556185 | UGP2 | NM_001001521 | -2.78 | 2.20E-04 | 1.16E-03 |
| 2792069 | NAF1 | NM_138386 | -2.69 | 4.82E-02 | 4.60E-02 |
| 2858134 | PDE4D | NM_001165899 | -2.52 | 2.46E-15 | 2.21E-02 |
| 2926323 | EYA4 | NM_004100 | -2.50 | 1.48E-04 | 1.04E-02 |
| 3988638 | LONRF3 | NM_001031855 | -2.41 | 4.93E-05 | 2.37E-02 |
| 3025740 | TMEM140 | NM_018295 | -2.36 | 5.50E-04 | 7.92E-03 |
| 2664209 | SH3BP5 | NM_004844 | -2.35 | 3.45E-03 | 1.28E-02 |
| 2591421 | TFPI | NM_006287 | -2.33 | 1.50E-02 | 3.40E-02 |
| 2587937 | CHRNA1 | NM_001039523 | -2.31 | 1.35E-18 | 1.89E-02 |
| 3205659 | SHB | NM_003028 | -2.30 | 4.60E-02 | 1.50E-02 |
| 2556017 | C2orf86 | NM_015910 | -2.21 | 1.38E-07 | 4.72E-04 |
| 2650393 | PPM1L | NM_139245 | -2.11 | 2.62E-03 | 5.90E-03 |
| 2989493 | MIOS | NM_019005 | -2.10 | 6.40E-04 | 3.03E-02 |
| 3519309 | SPRY2 | NM_005842 | -2.07 | 1.74E-02 | 1.42E-02 |
| 2711644 | ATP13A3 | NM_024524 | -1.92 | 4.19E-02 | 1.76E-02 |
| 3776504 | TGIF1 | NM_170695 | -1.85 | 3.20E-04 | 3.84E-02 |
| 3063856 | GATS | NR_028038 | -1.83 | 7.35E-03 | 3.20E-02 |
| 2930243 | SASH1 | NM_015278 | -1.78 | 6.26E-03 | 1.50E-02 |
| 2886174 | SLIT3 | NM_003062 | -1.77 | 1.54E-06 | 2.54E-02 |
| 3936256 | BCL2L13 | NM_015367 | -1.75 | 1.23E-03 | 7.18E-03 |
| 3269328 | ZRANB1 | NM_017580 | -1.75 | 3.04E-02 | 1.34E-03 |
| 2812690 | MAST4 | NM_001164664 | -1.75 | 3.30E-20 | 2.69E-02 |
