# Supplementary material for: Inflammation-Induced Acute Phase Response in Skeletal Muscle and Critical Illness Myopathy
Source: PLoS One. 2014 Mar 20;9(3):e92048. doi: 10.1371/journal.pone.0092048 (PMC3961297; doi:10.1371/journal.pone.0092048)
Supplement: Table S6 — Direct muscle stimulation identified weakness with a sensitivity of 80% and a specificity of 83.3%. (DOC) [file pone.0092048.s011.doc]

**Table S6**

**Direct muscle stimulation identified weakness with a sensitivity of 80% and a specificity of 83.3%.**

|  | **Patients with excitable**  **muscle membrane** | **Patients with non-excitable muscle membrane** | **Total number of patients** |
| --- | --- | --- | --- |
| **MRC score ≥ 4** | 5 | 1 | 6 |
| **MRC score < 4** | 3 | 12 | 15 |
| **Total** | 8 | 13 | 21 |
