# Supplementary material for: Inflammation-Induced Acute Phase Response in Skeletal Muscle and Critical Illness Myopathy
Source: PLoS One. 2014 Mar 20;9(3):e92048. doi: 10.1371/journal.pone.0092048 (PMC3961297; doi:10.1371/journal.pone.0092048)
Supplement: Methods S1 — Details about electrophysiological measurements, muscle biopsies, microarray analyzes and quantitative real-time PCR, the animal model of polymicrobial sepsis by cecal ligation and puncture surgery, mass spectrometry, immunohistochemistry and ELISA, and human and murine myoblast culture, RT-PCR, and immunofluorescence are provided. (DOC) [file pone.0092048.s012.doc]

**Methods S1.**

**Electrophysiological measurements.** Bedside electrophysiological measurements were performed as described recently . Briefly, muscle membrane excitability, referred to as compound muscle action potential after direct muscle stimulation (dmCMAP), in *tibialis anterior* was measured using a Keypoint 2 portable electromyograph/neurograph (Medtronic, Skovlunde, Denmark), as shown previously . A conventional stimulating surface electrode or, in case of edema, subdermal electrodes, were placed longitudinally to muscle fibers and proximal to the distal tendon insertion. Guided by muscle twitch, concentric needle electrodes were placed 30 to 50 mm proximal to each other in order to record dmCMAP. Serial electrophysiological testing with the first test at the day of study enrolment, and subsequently in 3 day intervals, was performed until pathologic findings or muscle weakness was detected. Thereafter, testing was repeated weekly. dmCMAP levels below 3 mV in at least one measurement before awakening were considered as being pathologic, and consistent with the electrophysiological diagnosis of CIM .

**Muscle biopsies.** For immunohistochemistry and metachromatic ATPase staining, muscle tissues were mounted and frozen under cryoprotection. Biopsy specimens were stored at -80°C. Frozen sections with a thickness of 6 µm for immunohistochemistry and 10 µm for routine histology were cut using a Leica cryotome. For histological analyzes by Haematoxylin & Eosin and Gomori-trichrome staining skeletal muscle biopsy specimens were fixed using 3.7% paraformaldehyde and processed.

**Microarray analyzes and quantitative real-time PCR.** For microarray analyzes, RNAs were extracted by RNA mini kit (Qiagen, Düsseldorf, Germany), and residual genomic DNA removed by DNAse I treatment (Qiagen). Biotinylated complementary RNAs (cRNA) from six control, four non-CIM and three CIM patients were synthesized and hybridized to HuEx-1_0-st-v2 human exon arrays (Affymetrix) according to the manufacturer’s protocol. Arrays were quantile-normalized with respect to the probe GC content using the Robust Multichip Averaging (RMA) algorithm of the Partek Genomic Suite 6.5 and the Affimetrix “full” probe set annotation, including cDNA-based annotations and *ab-initio* gene predictions (n=878,017 probe sets). Not or low expressed probe sets were removed (min. signal > 100), and the remaining probe sets used for an ANOVA analysis on averaged gene level with the grouping critical ill patients (n=3 CIM; n=4 non-CIM) vs. controls (n=6) using gender as a cofactor. The false discovery rate (FDR) multiple testing correction (5% FDR) resulted in a set of 1,841 significant differentially expressed transcripts. The top 30 genes by fold-change increased or decreased in critical illness were revealed in Tables S1 and S2, respectively. Further we investigated in exon expression interaction with the grouping control vs. CIM patients vs. non-CIM patients. 1,948 transcript clusters, underwent 5% FDR were tested on differential expressions between CIM vs. non-CIM patients using LSD test statistic. The top 30 genes by fold-change increased or decreased in CIM patients are listed in Tables S3 and S4, respectively.

For RT-PCR analyzes, total RNA was isolated from skeletal muscle biopsies using TRIzol® Reagent (Invitrogen) according to the manufacturer’s protocol. cDNA synthesis of 1 µg of RNA per sample was carried out by using the SuperScript® First-Strand Synthesis System for RT-PCR (Invitrogen). Real-time polymerase chain reaction (RT-PCR) was performed using either TaqMan® Universal PCR Mastermix (Applied Biosystems) with commercially available primer and probe sets for human TNF-α (Hs00174128_m1), human IL-6 (Hs00985639_m1), or SYBR® Green reagent (Applied Biosystems) and self-designed primers (for primer sequences see Table S1); PCR reactions were performed in a Step-One^TM^ Plus thermocycler (Applied Biosystems) as described recently using a cDNA standard curve . To correct for potential variances between samples regarding differences in mRNA extraction and reverse transcription efficiency, genes expression were normalized to the expression of the reference genes glyceraldehyde-3-phosphate dehydrogenase (*GAPDH*) or *beta-2-microglobulin* of the same samples .

**Animal model of polymicrobial sepsis by cecal ligation and puncture surgery.** Polymicrobial sepsis was induced by cecal ligation and puncture (CLP) surgery according to a published protocol . All animal procedures were performed in accordance with the guidelines of the Max-Delbrück Center for Molecular Medicine and the Charité Universitätsmedizin Berlin, and were approved by the Landesamt für Gesundheit und Soziales (LaGeSo, Berlin, Germany) for the use of laboratory animals (permit number G 0129/12) and followed the “Principles of Laboratory Animal Care” (NIH publication No. 86-23, revised 1985), as well as the current version of German Law on the Protection of Animals. CLP was performed on 12 weeks old male C57Bl6/N mice. Mice were anesthetized with isoflurane, placed on a heating pad to assure a constant body temperature at 37°C measured by a rectal probe. After shaving and disinfection of the skin a midline laparotomy was performed, and the cecum was exposed and ligated using a non-absorbable surgical suture (Ethicon 6-0). A 21-gauge needle was used to puncture the cecum once, and a small amount of cecum content was extruded. The cecum was then replaced into the abdominal cavity, and the incisions of the peritoneum and skin were closed with two separate layers of a surgical suture (Ethicon 6-0). Sham mice were treated identically except for the ligation and puncture of the cecum. All mice were resuscitated by an i.p. injection of 1 ml sterile, 37°C-prewarmed Ringer’s solution. Metamizole (200mg/kg body weight) applied twice daily s.c. was used as analgesic. The mice did not receive any antibiotics. Under these conditions, all CLP mice showed signs of severe illness, such as immobility, elevated body temperature, shaking, increased heart and respiratory rates, within 24h after induction of sepsis. CLP (n=5) and sham (n=5) mice were sacrificed 24h after surgery and *gastrocnemius plantaris* and *tibialis anterior* were harvested for analysis. For details on RNA extraction, reverse transcription and real-time RT-PCR please see above. For immunohistochemistry, muscle tissues from *gastrocnemius plantaris* muscles were dissected, mounted and frozen under cryoprotection. Muscle samples were stored at -80°C. Frozen sections with a thickness of 6 µm were cut using a Leica cryotome. For details on immunohistochemistry please refer to this point. Microdialysis of the *vastus medialis* of septic and sham mice was performed 24h after CLP or sham surgery. Shortly, mice were anesthetized with isoflurane, placed on a heating pad to assure a constant body temperature at 37°C measured by a rectal probe. After shaving and disinfection of the skin a microdialysis probe (CMA 20) was inserted into the *vastus medialis* and connected to a CMA 402 syringe pump. The interstitial compartment was dialysed against lactate-free Ringers-solution (E156, Serumwerk Bernburg AG, Bernburg, Germany). Following 1h of equilibration of the membrane dialysis was performed at a perfusion rate of 0.3µl/min for 3h. Proteins obtained from microdialysis were analyzed by mass spectrometry.

**Mass spectrometry.** Proteins obtained from microdialysis were converted to peptides by a two-step digestion protocol using endopeptidase lys-C and trypsin. The proteins were denatured in denaturing buffer (6M urea, 2 M thiourea, 20 mM HEPES, pH 8.0) and digested with endopeptidase Lys-C for 3h. Then the reaction was diluted four-fold with 50 mM ammoniumbicarbonate buffer and 50U trypsin was added. The reaction was incubated at room temperature over night. Peptides were separated on an 15 cm reverse-phase column (3 µm beads, Reprosil, Dr. Maisch HPLC, Ammerbuch-Entringen, Germany) using a 10-50% acetonitrile linear gradient on an easy-nLC system (Proxeon, Dreieich, Germany). Separated peptides were directly sprayed into a Velos-OrbiTrap mass spectrometer (Thermo scientific, Dreieich, Germany). The recorded spectra were analyzed using the MaxQuant software package (version 1.2.2.5) by matching the data to the International Protein Index (IPI) mouse database (version 3.84) with a false discovery rate (FDR) of 1%.

**Immunohistochemistry and ELISA.** Immunofluorescence staining on frozen histological sections from the *vastus lateralis* muscle was performed as reported recently using the anti-human-SAA1 (1:50, Abnova) as primary and the anti-mouse-IgG-AlexaFluor®488 (1:150, Invitrogen) as secondary antibody. Membrane staining was performed using the anti-human-laminin (1:100, Sigma Aldrich) as primary and the anti-rabbit-IgG-AlexaFluor®555 (1:150, Invitrogen) as secondary antibody. Immunohistological sections were embedded in ProLong® Gold Antifade Reagent (Invitrogen) containing 4',6-diamidino-2-phenylindole (DAPI) for nuclei stain. Immunofluorescence staining on frozen histological sections from the *gastrocnemius/plantaris* muscle of CLP and sham mice was performed as reported recently using the anti-mouse-SAA1 (1:50, R&D Systems) as primary and the anti-goat-IgG-FITC (1:100, Millipore) as secondary antibody. Immunohistological sections were embedded in ProLong® Gold Antifade Reagent (Invitrogen) containing DAPI for nuclei stain. Fluorescence microscopy was performed using the Leica CTR 6500 microscope and the Leica DFC 360 FX digital camera.

A commercially available enzyme-linked immunosorbent assay (HU SAA ELISA KIT, KHA0011 (Invitrogen)) was used to quantify A-SAA in serum samples of healthy controls (n=6), non-CIM (n=11) and CIM (n=19) patients according to manufacturer’s instructions.

**Human and murine myoblast culture, RT-PCR, and immunofluorescence.** Cell culture experiments of human (CHQ cells) and murine (C2C12 cells) myoblasts were performed as described recently . Purity of the human myoblast culture was analyzed by detection of the myocyte marker protein desmin using immunocytochemistry with an anti-desmin antibody (1:50, Chemicon). Differentiation of human myoblasts to myotubes was induced at confluence by replacing growth medium (M199 medium and DMEM high glucose (4.5 g/l glucose) (Sigma Aldrich), 1:4 ratio; supplemented with 20% FCS (Sigma Aldrich), 50 μg/ml gentamycin (Sigma Aldrich)) with differentiation medium (DMEM high glucose (4.5 g/l glucose). Differentiation of murine myoblasts to myotubes was induced at confluence by replacing growth medium (DMEM high glucose (4.5 g/l glucose) (Sigma Aldrich GmbH), supplemented with 20% FCS) with differentiation medium (DMEM low glucose (1 g/l glucose) supplemented with 2% FCS). At 5 days of differentiation, human myotubes were treated with solvent control (0.1% bovine serum albumin (BSA)), human recombinant IL-6 (100 ng/ml; Miltenyi Biotec), and/or human recombinant TNF-α (10 ng/ml; R&D Systems or Miltenyi Biotec) for 16 h. At 5 days of differentiation, murine myotubes were treated with solvent control (0.1% BSA), murine recombinant IL-6 (100 ng/ml; R&D Systems) or murine recombinant TNF-α (10 ng/ml; eBioscience) for 16 h. For RT-PCR analyzes, cells were collected and RNA was isolated using TRIzol® Reagent (Invitrogen) following manufacturer’s protocol. cDNA synthesis and RT-PCR were performed as described above, and *beta-2-microglobulin* or *glyceraldehyde-3-phosphate dehydrogenase* (*GAPDH*) were used as reference genes. Primer sequences are provided in Table S5.

For immunocytochemistry, CHQ cells differentiated for 5 days were treated for 16h with either 1 µg/ml of lipopolysaccharides (LPS, *E. coli*, Sigma Aldrich), 10 ng/ml human recombinant TNF-α, 100 ng/ml IL-6 (Miltenyi Biotec), or both cytokines together, or solvent control (0.1% BSA in 1x PBS). C2C12 cells were differentiated for 5 days and treated for 16h with either 1 µg/ml of LPS (*E. coli*, Sigma Aldrich), 10 ng/ml murine recombinant TNF-α (eBioscience) , 100 ng/ml murine recombinant IL-6 (R&D Systems), or both cytokines together, or solvent control (0.1% BSA in 1x PBS). Differentiated CHQ and C2C12 cells were fixed with 3.7% paraformaldehyde for 10 min at room temperature, permeabilized with 0.2% Triton-X-100 in 1x PBS for 15 min at room temperature, and blocked with 1% BSA for 1 hr at room temperature. In CHQ cells SAA1 was detected with the anti-human-SAA1 (1:50, Abnova) as primary and the goat-anti-mouse-IgG-AlexaFluor®488 (1:50, Invitrogen) as secondary antibody. In C2C12 cells SAA1 was detected with the anti-mouse-SAA1 (1:50, R&D Systems) as primary and the anti-goat-IgG-FITC (1:100, Millipore) as secondary antibody. Stained cells were embedded in ProLong Gold® Antifade Reagent that contained DAPI for nuclei stain (Invitrogen). Immunostaining was analyzed using the Leica CTR 6500 microscope and the Leica DFC 360 FX digital camera.

**References**

1. Weber-Carstens S, Koch S, Spuler S, Spies CD, Bubser F, et al. (2009) Nonexcitable muscle membrane predicts intensive care unit-acquired paresis in mechanically ventilated, sedated patients. Crit Care Med 37: 2632-2637.

2. Trojaborg W, Weimer LH, Hays AP (2001) Electrophysiologic studies in critical illness associated weakness: myopathy or neuropathy--a reappraisal. Clin Neurophysiol 112: 1586-1593.

3. Khan J, Harrison TB, Rich MM, Moss M (2006) Early development of critical illness myopathy and neuropathy in patients with severe sepsis. Neurology 67: 1421-1425.

4. Benjamini Y, Hochberg Y (1995) Controlling the false discovery rate: a practical and powerful approach to multiple testing. Journal of the Royal Statistical Society: Series B 57: 289-300.

5. Fielitz J, Hein S, Mitrovic V, Pregla R, Zurbrugg HR, et al. (2001) Activation of the cardiac renin-angiotensin system and increased myocardial collagen expression in human aortic valve disease. J Am Coll Cardiol 37: 1443-1449.

6. Fielitz J, Kim MS, Shelton JM, Latif S, Spencer JA, et al. (2007) Myosin accumulation and striated muscle myopathy result from the loss of muscle RING finger 1 and 3. J Clin Invest 117: 2486-2495.

7. Fielitz J, Kim MS, Shelton JM, Qi X, Hill JA, et al. (2008) Requirement of protein kinase D1 for pathological cardiac remodeling. Proc Natl Acad Sci U S A 105: 3059-3063.

8. Bierbrauer J, Koch S, Olbricht C, Hamati J, Lodka D, et al. (2012) Early type II fiber atrophy in intensive care unit patients with nonexcitable muscle membrane. Crit Care Med 40: 647-650.

9. Rittirsch D, Huber-Lang MS, Flierl MA, Ward PA (2009) Immunodesign of experimental sepsis by cecal ligation and puncture. Nat Protoc 4: 31-36.

10. Cox J, Mann M (2008) MaxQuant enables high peptide identification rates, individualized p.p.b.-range mass accuracies and proteome-wide protein quantification. Nat Biotechnol 26: 1367-1372.

11. Kim MS, Fielitz J, McAnally J, Shelton JM, Lemon DD, et al. (2008) Protein kinase D1 stimulates MEF2 activity in skeletal muscle and enhances muscle performance. Mol Cell Biol.

12. Mamchaoui K, Trollet C, Bigot A, Negroni E, Chaouch S, et al. (2011) Immortalized pathological human myoblasts: towards a universal tool for the study of neuromuscular disorders. Skelet Muscle 1: 34.
